# Evaluation and Analysis of Water Quality of Marine Aquaculture Area

**DOI:** 10.3390/ijerph17041446

**Published:** 2020-02-24

**Authors:** Xianyu Zhang, Yingqi Zhang, Qian Zhang, Peiwu Liu, Rui Guo, Shengyi Jin, Jiawen Liu, Lei Chen, Zhen Ma, Ying Liu

**Affiliations:** 1 Dalian Ocean University, College of Marine Sciences, Dalian 116023, China; 15040168681@163.com (X.Z.); yingqi_77@163.com (Y.Z.); l532971378@163.com (P.L.); ruisweet@163.com (R.G.); kimziho@outlook.com (S.J.); 15124137622@163.com (J.L.); chenlei@dlou.edu.cn (L.C.); mazhen@dlou.edu.cn (Z.M.);; 2 Key Laboratory of Environment Controlled Aquaculture, Ministry of Education, Dalian Ocean University, Dalian 116023, China

**Keywords:** water quality assessment, principal component analysis, antibiotic resistance genes, marine aquaculture, salinity, dissolved oxygen

## Abstract

In the rapid development of marine aquaculture, the water quality of aquatic environments is regarded as a main limiting factor. Therefore, it is necessary to assess the water quality and environmental conditions in marine aquaculture areas and find out the main influencing factors regarding damage to the water quality environment. In the present research, pond aquaculture and cage aquaculture areas were sampled in May, August and November in 2018. Nine water quality indicators were detected, including pH, temperature, salinity, dissolved oxygen, molybdate-reactive phosphorus, chemical oxygen demand, chlorophyll a, inorganic nitrogen and antibiotic resistance genes (ARGs). Principal component analysis (PCA) was used to analyze the water quality conditions, spatial–temporal changes, and the driving factors in pond and cage aquaculture areas. The results showed that three main components were extracted from the pond aquaculture area, which explained 66.82% of the results, the most relevant factors are salinity, dissolved oxygen and ARGs. For the cage aquaculture area, three main components were extracted which can account for 72.99% of the results, the most relevant factors are chlorophyll a, salinity and dissolved oxygen. The comprehensive scores of the principal components indicated that the heaviest polluted months in pond and aquaculture areas were August and November, respectively. The water quality of the pond aquaculture area is mainly limited by the volume of the pond, while aquaculture activities and seasonality are the main factors for cage aquaculture. ARGs in cage culture areas showed more variety and frequency compared with pond culture areas, which indicated that terrestrial input might be one of the sources for ARGs occurrence. The results would be helpful for the relevant authorities to select water quality monitoring parameters in marine aquaculture areas.

## 1. Introduction

In recent years, with the rapid development of marine aquaculture in China, the increasing scale and breeding density of aquaculture have induced a frequent occurrence of diseases and environmental pollution [1]. A large amount of the nitrogen and phosphorus in feeds cannot be utilized by fish during aquaculture, which makes the nutrient concentration higher in aquaculture areas than in other natural areas [2]. The nutrient input from aquaculture effluent may cause a deterioration of water quality in the aquatic environment [3]. Besides, aquatic product quality is easily influenced by the condition of the water environment [4]. Some antibiotics are considered as “pseudo-persistent” contaminants due to their continuous release and lower degradability in the environment. The persistent organic pollutants in aquatic environment can be enhanced through biological amplification, which may pose a potential hazard to human health. Moreover, the abuse of antibiotics in aquaculture leads to the emergence of drug resistance in aquatic animals, which arouses serious clinical and public health problems on a global level [5,6].

China is the largest producer and user of antibiotics in the world; based on a survey in 2013 [7], veterinary antibiotics consumed 84,240 tons (52%) of the total antibiotics’ usage in China. Antibiotic residues have been detected at a high level in various environmental matrices. In the research of Zhou et al. [8], it was found that 25 antibiotics were detected in the Yangtze River, with a total concentration of 0.22–366 ng/L. A variety of antibiotics were widely detected in Dalian coastal waters, with concentrations ranging from 22.6 to 2402.4 ng/L [9]. The residual antibiotics in the environment could not only induce adverse effects on the nontarget organisms but also are considered as one of the selected stresses for the occurrence of the promotion of bacterial antibiotic resistance. It has been demonstrated that the frequent use of antibiotics in the animal husbandry industry as well as in aquaculture is one of the critical reasons for the high abundance of antibiotic resistance genes (ARGs) [10]. Usually, antibiotics are applied through feed addition in aquaculture; only about 25% antibiotics will be absorbed by aquatic animals, while the excess unabsorbed antibiotics or antibiotics not ingested by fishes will be discharged in the form of urine and feces, then directly dissolved/degraded in water by bacterial biomass, which eventually will come back to aquatic environment. The long-term existence of antibiotics leads to the accumulation of antibiotic-resistant bacteria and ARGs, which may pose a risk of transmission to the general population and to wildlife, causing treatment-resistant illness [11,12]. 

Zhuanghe, located in the southeast of Liaodong Peninsula, is an important marine aquaculture area in Dalian in China. It has a coast length of 285 km and culture area of 620 km^2^, with over than 190 aquaculture companies around. The main aquaculture species included sea cucumber (*Stichopus japonicus*), clam (*Meretrix meretrix L*), scallop (*Patinopecten yessoensis*), oyseter (*ostrea gigas thunberg*), shrimp (*Penaeus orientalis*), puffer (*Takifugu rubripes*), etc. Pond culture, cage culture, floating raft culture and indoor industrial culture are the main culturing models. Among them, pond culture is mainly carried out in enclosed ponds constructed with dams by the sea, and the exchange of water relies on the regularity of the tide. A positive aspect is that pond culture is convenient to manage with a relatively small water volume. On the other hand, water exchange is restricted by the water quality from the sea and is vulnerable to being affected by the water environmental conditions, such as organic pollutants, nutrients, temperature, dissolved oxygen and so on [13]. Cage and floating raft cultures are characterized in high-breeding density and high-profit areas and use metal or synthetic fibers to construct the cage or raft; however, these methods are susceptible to waves, temperature and other factors [14]. In recent years, marine water quality has deteriorated due to continuously discharging wastewater from industry and land domestic sewage, as well as untreated effluent from aquaculture. As a consequence, the quality and the yield of aquatic products significantly declined, while the source of contaminants was difficult to determine, which affected the implementation of pollution control. Therefore, it is of great significance to monitor the water quality and make a comprehensive evaluation in aquaculture areas [15].

Currently, the commonly used methods in marine environmental assessment for water quality classification include single factor assessment and multivariate analysis. Single factor assessment is a simple and convenient method for water quality classification, judged according to the most impaired assessment factor and compared to the corresponding water quality standard [16]. The multivariate analysis includes principal component analysis (PCA), factor analysis (FA), the fuzzy comprehensive evaluation method and integrated analysis method, etc. [17]. Compared with single factor assessment, PCA has the advantages of keeping the original information and uncorrelated variables at the same time, with the aim of screening out the independent principal component factors through dimension reduction analysis [18,19]. To effectively address the water quality impairment and pollutant sources, PCA has been extensively used in the evaluation of water quality, eutrophication and pollution degree [20]. At present, there are many studies on the analysis of water quality by PCA [21,22]. In the study by Ouyang, PCA was verified as a useful tool for the identification of important surface water quality monitoring stations and water quality parameters [23]. Besides, PCA was also used in the selection of water quality parameters for the water quality index (WQI), and the number of monitoring parameters was reduced from 28 to 9 after the application of PCA [24]. To ensure the objectivity of results and the effectivity of resources, the local conditions should be considered to reevaluate the PCA results. At the same time, in order to improve the method of water quality assessment, the role of principal component analysis still needs to be compared with other water quality assessment analysis methods that can reduce the workload by reducing the indicators [25].

Although emerging pollutants in the aquatic environment have been frequently detected, they were not included in the scope of water quality monitoring. The types of pollutants varied among different aquaculture models, which affected the distribution and transfer of ARGs in the environment. At present, there is no related research on water quality assessment with ARGs and other water quality parameters. In the present study, principal component analysis was used to assess the temporal and spatial variations of water quality in pond and cage culture areas and to analyze the driving factors among various water quality parameters and the ARGs detection rate in the different aquaculture areas. The results are expected to provide a valuable method for the evaluation of impaired water environments, especially for areas with fixed pollutant input. 

## 2. Materials and Methods 

### 2.1. Sampling Sites and Time

**Figure 1:**
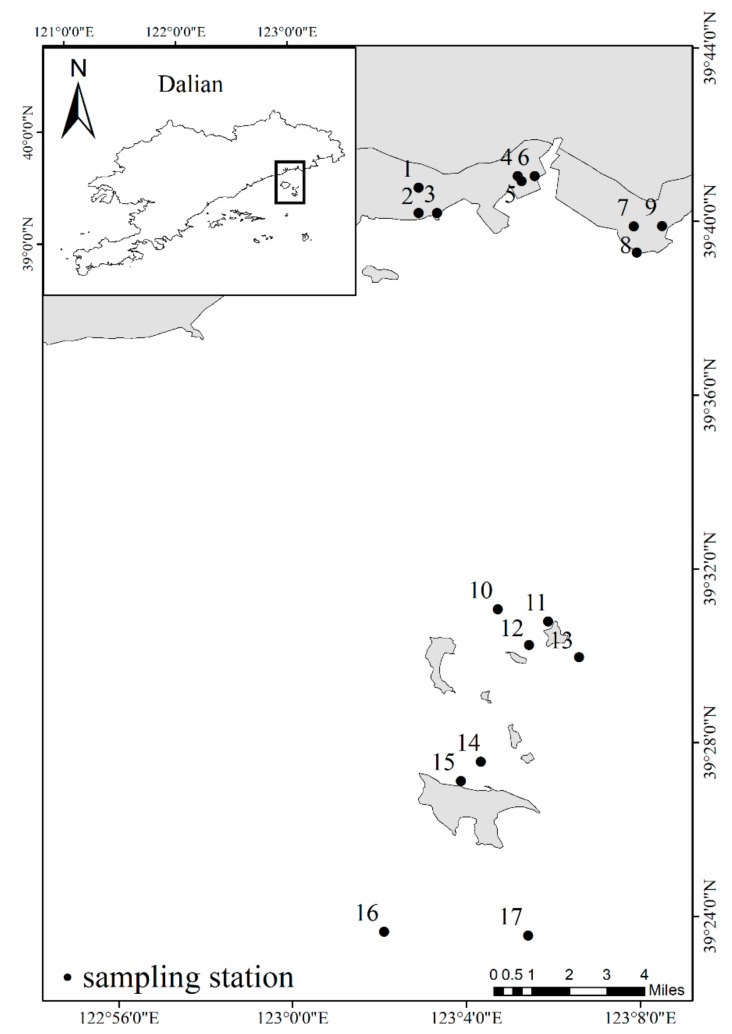
Sampling sites in the marine aquaculture area of Zhuanghe.

The sampling sites covered both pond culture and cage culture areas in a typical district of marine aquaculture in Zhuanghe. There are nine culture ponds (1–9) featuring the breeding of sea cucumber on the cofferdam shore, and 10 points (10–17) around cage or floating raft with fish and shellfish cultures distributed 15–30 km away from the mainland. The water outside the ponds and cages came from the same source, and water quality differences and effects between them were ignored in the present study. Detailed sampling site information is shown in Figure 1. Considering the growth cycle of cultured species and the fishery activities, samplings was conducted in May, August and November in 2018, which represented the growth, breeding and harvest period of aquaculture organisms, respectively. For sea cucumber culture ponds, water was exchanged depending on the tide once or twice per month. Sea cucumber seedlings were cultured in sampling sites 1–3, while adult sea cucumber was cultured in sampling sites 4–9. For cage culture areas, the bivalves *Argopecten irradias*, *Chlamys farreri*, *Crassostrea gigas* and *Scapharca broughtonii* were the main breeding species; thereinto fish net cages for *Seriola aureovittata* breeding were settled near sampling sites 14, 16 and 17. The water depths of the sea cucumber cultural ponds were about 2.0 m, and those of the cage culture area were in the range of 8.6–24.8 m.

### 2.2. Sampling and Chemical Analysis

The sampling, preservation, transportation and detection of water samples were strictly performed following the National Specification for Marine Monitoring standard methods (GB 17378.4-207) (SEPA, 2002). Water samples were taken at depth of 0.5 and 3.0 m in the pond and cage culture areas, respectively. The parameters of pH (± 0.2), temperature (T, ± 0.02 °C), salinity (SAL, ± 0.1 ppt), dissolved oxygen (DO, ± 0.01 mg/L) were detected by a multi-parameter water quality analyzer (ProPlus, YSI, Yellow Springs, OH, USA) at the sampling sites. Molybdate-reactive phosphorus (MRP) was determined by the methods of phosphor molybdenum blue spectrophotometry. Under acid conditions, molybdic acid reacts with inorganic phosphorus to form yellow ammonium phosphomolybdate, which is then reduced to dark blue molybdenum blue by the original agent. The absorbance was measured at 650 nm by spectrophotometer, and the content of inorganic phosphorus was calculated by a standard curve. Chemical oxygen demand (COD) was determined by the method using alkaline potassium permanganate, which is not affected by salinity [26]. Under the condition of alkaline heating, we oxidized the oxygen demand substance in seawater with a known amount of potassium permanganate. Under sulfuric acid conditions, excess potassium permanganate and manganese dioxide were reduced by potassium iodide. The free iodine was titrated with sodium thiosulfate standard solution. The volume of sodium thiosulfate consumed was determined by a formula and known concentration. Chlorophyll a (Chla) content was determined by Dimethyl Formamide (DMF) extraction methods. The chlorophyll was extracted by DMF, which could be directly determined according to the absorbance after being kept away from light for 12 h [27]. Dissolved inorganic nitrogen (DIN, the sum of ammonium nitrogen (NH^4+^-N), nitrate nitrogen (NO^3-^-N) and nitrite nitrogen (NO^2-^-N)) were determined by an automatic continuous flow analyzer (AA3, SEAL) [28]. In order to detect the emergence of ARGs in aquaculture wastewater, 1500 mL water samples were filtered by a 0.22 μm filter membrane; then, DNA was extracted by DNA extraction kit (Chengdu Foregene Biotechnology Co., Ltd, Chengdu, China). Seven ARGs (*flor, sul1, sul2, tetB, tetM, qnrS* and *ermB*) from five classes of commonly used antibiotics in aquacultures were selected. A Veriti™ 96-Well Thermal Cycler (Applied Biosystems™, ThermoFisher Scientific, Waltham, MA, USA) was employed using 2 × EasyTaq® PCR SuperMix (Beijing Transgen Biotech, Beijing, China) to determine the expression of ARGs. The primer sequences of ARGs are detailed in Table 1. According to the results of questionnaires, no records of antibiotic application in the process of aquaculture were found. 

### 2.3. Data Processing and Principal Component Analysis

The statistical analysis was based on the standardized sample matrix (19 sites × 3 phases × 9 variables) and summarized in Table 2, which presented the mean values ± SD of the results. To avoid misclassification due to wide differences in data dimensionality, data was standardized through z-scale transformation before applying PCA [36]. For the detection rate of AGRs, there is no exact standard range of AGRs that can be used as an index to analyze the other water quality factors. In the present study, when ARGs were detectable, the result was regarded as 1; otherwise, it was regarded as 0. The data were normalized according to the following formula:

(1) $$Z_{ij}=\frac{X_{ij-\mu }}{\sigma }$$

where $Z_{ij}$ represents the normalized value of the i-th station’s j index; $X_{ij}$ represents the measured value of the i-th station’s j indicator; μ represents the mean value of variables; and σ represents the standard deviation of variables.

In this study, all the data statistical analysis was conducted by SPSS 18.0, KMO and Bartlett’s sphericity test were used to verify the applicability of PCA to raw data [37]. Then, the corresponding correlation coefficient matrix, eigenvalues and eigenvectors were calculated. We adopted the principle that the eigenvalue > 1.0 to determine the number of principal components m [21]. The maximum variance was used to normalize the rotation of the original data to rotate the factor [38].

To estimate the degree of contamination, the common factor for each sample needed to be calculated, and the principal component composite score could be used both for model diagnosis and as raw data for further analysis. Firstly, the values of the first m principal components of each sample were calculated as the following formula:

(2) $$F_{ig}=Z_{i1}l_{g1}+Z_{i2}l_{ig}+Z_{ig}l_{ig}+\cdots +Z_{ig}l_{ig}i=1,2,\cdots ,n;g=1,2,\cdots ,m.$$

Then, the weighted summation was performed according to the first m principal components, and the weight coefficient of each principal component was the variance contribution rate of the principal component, i.e., $\lambda_{g}/\sum_{g=1}^{m}\lambda_{g}$. For the sample i, the comprehensive score of each month’s standing was calculated using the following formula:

(3) $$F_{i}=\sum_{g=1}^{m}\left(\lambda_{g}/\sum_{g=1}^{m}\lambda_{g}\right).$$

The weighted sum of the previous three principal components was used to get the comprehensive scores of each sample, by which the Box-plot and the sampling sites’ comprehensive score maps were made. Spearman’s rank correlation coefficient analysis was used to analyze the relevance between factors and the principal components, and we obtained the main driving factors [39]. 

## 3. Results 

### 3.1. Water Quality Parameter Detection

As shown in Table 2, nine water quality parameters were summarized as mean value ± standard deviation. During the entire detected period, no abnormal raw data for all the parameters were found. The values of pH varied in the range of 7.9–8.4, and no significant differences were observed between seasons and areas. The values of temperature varied in the range of 9.8–24.7 ℃, and the highest water temperature of the pond culture area was shown in May and in August for the cage culture area. The values of SAL in the two aquaculture areas varied in the range of 28.5–32.1 ppt, while the lowest and highest values of SAL were shown in the pond culture area and kept relatively stable in cage culture area. Dissolved oxygen (DO) values varied greatly with seasons, ranging from 7.3 to 10.4 mg/L (DO saturation index 77.18%–99.30%). The contents of Chla varied in the range of 0.43–6.86 μg/L. The amount of COD, MRP and DIN varied in range of 0.56–11.12 mg/L, 0.008–0.328 mg P/L and 0.002–1.796 mg N/L, respectively. Compared with the water quality standard, the dissolved oxygen (<6 mg/L), molybdate-reactive phosphorus (<0.045 mg P/L) and inorganic nitrogen (<0.2 mg N/L) in the present study meet the class I of water quality standard, while contents of COD (> 5 mg/L) exceeding the standard were observed in August in the pond culture area. In contrast, DO, MRP and COD in the cage culture area did not exceed the standard for the entire monitored month.

To further explore the difference of ARGs distribution in different sources, the detection rates of seven ARGs in pond and cage culture areas were analyzed. The detection rates of ARGs varied greatly ranging from 11%–100%, and relatively low detection rates of ARGs were observed in the pond culture area. Six kinds of ARGs were detected in the pond aquaculture area; among them, the detection rates were 11% and 94% for sulfonamides ARGs (*sul1* and *sul2*), 5% for chloramphenicoles ARGs (*flor*), 6% and 0% for tetracyclines ARGs (*tetM* and *tetB*), 11% for quinolones ARGs (*qnrS*) and 44% for macrolides ARGs (*ermB*). In the cage culture area, the detection rates of sulfonamides ARGs (*sul1* and *sul2*) and chloramphenicoles ARGs (*flor*) were 100%, those for tetracyclines ARGs (*tetM* and *tetB*) were 81% and 57%, quinolones ARGs (*qnrS*) and those for macrolides ARGs (*ermB*) were 87% and 82%, respectively.

### 3.2. Water Quality Assessment by PCA

Principal component analysis was performed on the data matrix of variables from pond and cage marine aquaculture areas in Zhuanghe using the computed eigenvalues and weights of parameters. After the verification of the data’s validity by Bartlett’s sphericity test (<0.001) and KMO test for pond and cage culture, the nine parameters yielded three principal components (eigenvalues > 1) explaining sample variances of about 66.82% and 72.99% for pond and cage culture areas, respectively (Table 3). Since pond and cage culture areas were combined to calculate the correlation matrix, the correlation coefficients should be interpreted with caution as they are simultaneously affected both by spatial and temporal variations. Various factor loadings in principal components are shown in Table 4. For the pond culture area, PC1 explained 31.79% of total variance, which had positive loadings (> 0.70) on COD (0.72) and Chla (0.74) and significant negative loadings on SAL (–0.86). In the cage culture area, pH (0.71), T (–0.76) and Chla (0.80) occupied higher loading in PC1 (> 0.70).

### 3.3. Spatial and Temporal Variations of Water Quality

**Figure 2:**
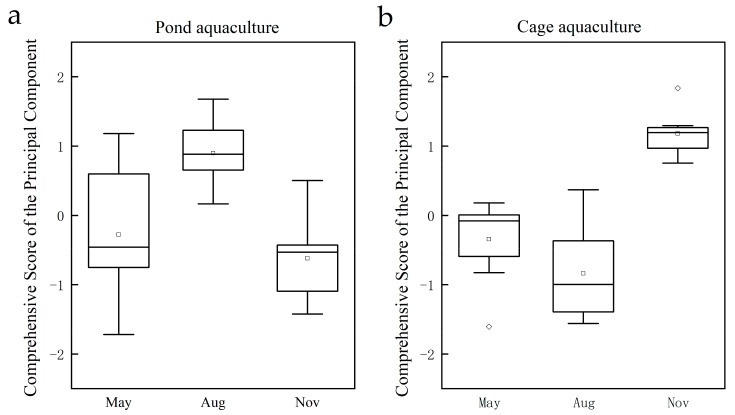
Temporal distribution of comprehensive scores. (**a**) Pond aquaculture area; (**b**) cage aquaculture area. Note: the top, bottom and middle lines of the Box plot represent the upper and lower quartiles and the median, respectively; the vertical part of the box body extending upward and downward represents the range of data distribution.

Box and whisker plots of the comprehensive score of principal components were constructed to evaluate water quality in different aquaculture patterns associated with temporal variations (Figure 2). The higher the comprehensive scores of principal components, the more serious the water pollution. In the pond culture area, the highest comprehensive score of principal components was in August (0.88), then in May (–0.46), and lowest in November (–0.53). In the cage culture area, the comprehensive score was highest in November (1.19), second in May (–0.08), and lowest in August (–1.00). 

**Figure 3:**
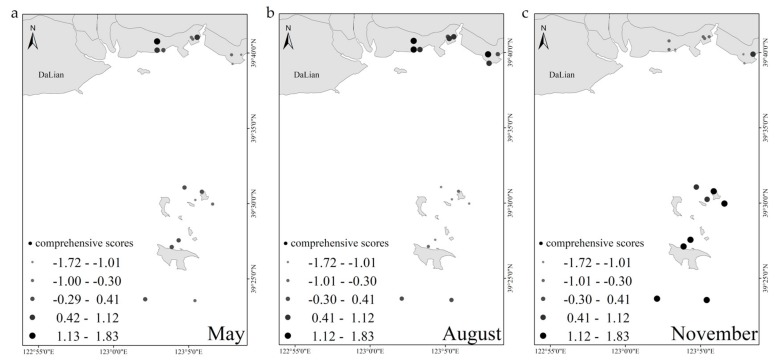
Spatial distributions of the comprehensive scores of principal components in aquaculture area. (**a**) Principal component comprehensive score in May; (**b**) principal component comprehensive score in August; (**c**) principal component comprehensive score in NovemberDriving Factors Analysis

The spatial distributions of comprehensive scores in different months in the pond culture area and cage culture area of Zhuanghe are shown in Figure 3. Obvious spatial distribution trends in different culture stages were observed. In May and August, comprehensive scores in most sampling sites of the pond culture area were significantly higher than that in the cage culture area. In contrast, comprehensive scores in the pond culture area were significantly lower than in the cage culture area in November. 

Principal component analysis is an effective tool to figure out the driving factors of water pollution. In order to extract the dominant factors from numerous physicochemical parameters related to water quality, the driving factors of water pollution in different marine aquaculture areas were analyzed. In the present study, a Spearman’s rank correlation coefficient analysis between the principal components and parameters was carried out followed with the research of Lundberg et al. [39]. As shown in Table 5, SAL, DO and ARGs were the main driving factors of PC1, PC2 and PC3 (*P* < 0.01) in the pond culture area, while Chla, SAL and DO were the main driving factors of PC1, PC2 and PC3 (*p* < 0.01) in cage culture areas.

## 4. Discussion

PCA is useful when several related random environmental variables are simultaneously considered. The nine original variables were reduced to three key independent factors that influenced water quality in pond and cage culture areas. In the pond culture area, PC1 had high loadings on SAL, Chla, and COD, which represents the contribution of phytoplankton growth from the natural impacts on the water condition, such as the water exchange rate of pond and rainfall. In early spring, when the ice begins to melt, fluctuations in salinity affect cultured organisms. Even though algae have definite selectivity and tolerability to salinity variability [40], their community structure is still easily influenced by salinity fluctuations [41]. COD served as an important parameter in traditional water quality monitoring to reflect the organic matter in marine waters. To guarantee the rapid growth of sea cucumber, herbicide may be used to suppress the outbreak of macroalgae in sea cucumber culture ponds, and the death of macroalgae may cause the increase of COD [42]. PC2 had high loadings on temperature and DO, which can be regarded as the index of aquaculture organism’s growth conditions in pond culture. Temperature and dissolved oxygen are the critical factors in the process of aquaculture [43], which presented significant influences regarding the growth and development of aquaculture organisms. PC3 had high loadings on DIN and ARGs, which was identified as human activity-induced pollution. Nitrate in ponds is mainly derived from aquaculture activities and wastewater discharges [44], and ARGs occurrences mainly came from microorganisms after antibiotic administration in ponds [45,46,47]. In the cage culture area, PC1 had high loadings on Chla, T, pH which can be regarded as the photosynthesis of phytoplankton. Temperature affects phytoplankton growth by affecting photosynthesis and phosphorus concentration [48], and pH affects the photosynthesis of phytoplankton by affecting fluorescence absorption of chlorophyll [49].In PC2, SAL with positive loading and DIN with negative loading occupied relative high loadings. Salinity variations led to the changes of microbial function and the process of nitrification and denitrification, affecting the contents of DIN in water [50]. Therefore, PC2 can be regarded as the microorganism changes in seawater. In PC3, DO showed a high loading.

Different trends in the comprehensive score box of the pond culture and cage culture were observed, and the season-correlated parameter can be taken as representing the major source of temporal variations in water quality. In the summer of 2018 in Dalian, the abnormally high temperature caused the death of many aquaculture organisms, which may explain the highest comprehensive score of principal components in August in the pond culture area. The volume of water in the pond culture area was limited and easily influenced under high temperature, which would aggravate the pollution and result in a high comprehensive score in August. In the cage culture area, the comprehensive score was highest in November and lowest in August. As regards the better water exchange ability in the cage culture area, high temperature was not the critical factor in the impairment of water quality in August. Moreover, the light intensity and illumination period was suitable for the growth of phytoplankton, which accordingly induced the decrease of nutrient concentration in seawater. In November, the water temperature decreases, which is regarded as one of the factors leading to the decline of primary productivity [51]. Fishery harvest activities and the frequent human activities were also considered as critical reasons for high comprehensive scores and heavy pollution during this period in the cage culture area. 

The spatial analysis revealed a significant difference between pond and cage culture areas. April and May are the important cultivation period for sea cucumber seedlings. To achieve the rapid growth rate, soybean and eelgrass (*Zostera marina*) were fed as the supplemental feeds, the remnants of which may cause a deterioration of water quality and elevate the comprehensive scores of principal components. In cage culture, sampling sites 16 and 17 exhibited high-level contaminants, in which *Seriola aureovittata* were cultured in fish net cages intensively. The extreme weather played a direct role in the ponds with shallow water depth, which caused the death and hypoxia of aquaculture organisms, as well as the outbreak of epidemic disease. In November, the opposite phenomenon for the scores of the cage culture area was observed, which was significantly higher than that of the pond culture area. This may be due to the pollution degree of the seawater in cage culture areas, which was increased by the influence of human fishery harvest activities and fewer sea cucumber activities in pond culture under low temperature conditions. Considering the descriptions above, the difference in aquaculture water quality was mainly affected by the species and the aquaculture patterns.

The common driving factors in pond and cage culture areas are SAL and DO. Salinity is a key factor in the process of aquaculture and showed a negative correlation in the pond culture area and positive correlation in the cage culture area. From the perspective of original data, the salinity varied within the normal range (28.2–33.0), with the variations probably due to the sudden weather changes [52]. It was found that, in August, Dalian was often affected by heavy rainfall. A cultural pond is a half-open artificial ecological system, which is susceptible to the disturbance of abnormal natural conditions. Heavy rainfall brought a huge amount of freshwater injection and disturbed the sediment in the pond, and the salinity sharply decreased which gave fierce fluctuations in bacteria communities and oxygen concentrations [53], which may also have resulted in the death of sea cucumbers [54]. Thus, this showed the serious pollution and negative correlation in the area with low salinity.

For the cage culture area, rainfall could bring oxygen and other substances and promote the growth of plankton, which is conducive to the absorption of nutrients. Besides, DO is also a crucial indicator which not only directly affects the growth of cultured organisms but also is vital to the decomposition of harmful substances at the bottom of the pond. It takes 4570 g of oxygen to oxidize 1000 g of ammonia to nitrate; the nitrification reaction was blocked under the low DO concentration. The decrease of DO led to the increase of CO_2_ content and the decrease of the pH value. It has been reported that the oxygen consumption of oxidation decomposition accounts for 40% of the total oxygen consumption in the aquaculture pond, while only 12% was consumed for aquatic organisms, even though this was less acute in seawater than in freshwater [55]. In both cultural areas, DO presented a positive correlation, which indicated that the water quality was better in the area with a high concentration of dissolved oxygen. In aquaculture, DO is influenced by the temperature and density of cultural organisms; it dropped significantly when the temperature and breeding density increased. In addition, Chla showed a positive correlation in the cage culture area. Chla was considered as the key driving factor in the culture area which could represent the density of phytoplankton, which contributed to the utilization of the redundant nutrition. The abundance of Chla indicated the risk of eutrophication, while the weakened sunlight may have decreased the photosynthesis of algae and the production of oxygen. Furthermore, oxygen in the water was to a great extent consumed by the degradation of organic matter from dead phytoplankton cells, which may aggravate the water pollution. Furthermore, ARGs showed a negative correlation in the pond culture area. ARGs were considered as an emerging contaminant in the aquatic environment due to their potential risk. It was reported that 11 ARGs with frequent presence were observed in the sediments of the East China Sea bays, and sulfonamide resistance was the most prevalent ARG type [12]. Consistently, sulfonamide resistance was also detected at a high rate both in pond and cage culture areas. ARGs in the cage culture area showed more variety and frequency compared with the pond area, which indicated the terrestrial input might be one of the sources for ARGs occurrence, such as hospitals, land farms and wastewater treatment plants. ARGs can be widely transferred among bacterial species in the various environmental media. Even no record of antibiotics application in aquaculture, the residues of heavy metals, micro plastics and other organic pollutants in the aquatic environment may promote the transfer of ARGs [56]. Therefore, the risk assessment and removal of ARGs in aquaculture wastewater should be taken into consideration.

## 5. Conclusions

Water quality assessment with ARGs detection rate and other water quality parameters between different culture models was investigated in 19 sampling sites in the Zhuanghe marine aquaculture region. The PCA results suggest that three principal components explained 66.82% and 72.99% of the results in pond and cage culture areas. According to the time–space analysis of principal component comprehensive scores, August and November were the most polluted months in pond and cage culture areas, and seasonal variation and human fishery harvest activities affect the pollution of these two different types of aquaculture areas seriously. Further analysis showed that Chla, SAL, DO and ARGs are the main driving factors. Because of the limited area of the pond, weather changes exerted critical impacts on its water quality, while the water quality in the cage culture was mainly related to human fishery activities. In the present study, the detection rates of ARGs were varied in pond and cage culture areas. To explore the reason for the occurrence of ARGs in aquaculture, environmental stresses such as antibiotics, heavy metals, etc., should be included in future research.

## Figures and Tables

**Table 1:** Primer sequences of antibiotic resistance genes (ARGs).

ARGs Name	Primers Sequences (5’-3’)	Product Length (bp)	Annealing Temperature	Reference
*flor*	F: TATCTCCCTGTCGTTCCAG R: AGAACTCGCCGATCAATG	399	58 °C	[29]
*sul1*	F: CACCGGAAACATCGCTGCA R: AAGTTCCGCCGCAAGGCT	159	60 ℃	[30]
*sul2*	F: TCATCTGCCAAACTCGTCGTTA R: GTCAAAGAACGCCGCAATGT	105	56 ℃	[31]
*tetB*	F: CGAAGTAGGGGTTGAGACGC R: AGACCAAGACCCGCTAATGAA	192	56 ℃	[32]
*tetM*	F: ACAGAAAGCTTATTATATAAC R: TGGCGTGTCTATGATGTTCAC	171	60 ℃	[33]
*qnrS*	F: ACGACATTCGTCAACTGCAA R: TAAATTGGCACCCTGTAGGC	417	56 ℃	[34]
*ermB*	F: GATACCGTTTACGAAATTGG R: GAATCGAGACTTGAGTGTGC	364	58 ℃	[35]

**Table 2:** Water quality variables in pond and cage culture areas during different sampling seasons.

Sampling Type	Month	pH	T (°C)	SAL	DO (mg/L)	MRP (mg P/L)	COD (mg/L)	Chla (μg/L)	DIN (mg N/L)	ARGs (%)
Pond	May	8.41 ± 0.45	26.0 ± 0.7	31.3 ± 0.8	7.44 ± 1.44	0.095 ± 0.061	3.0 ± 1.5	2.03 ± 1.60	0.100 ± 0.086	22% ± 11%
	Aug	8.13 ± 0.23	20.7 ± 0.5	28.90 ± 0.53	8.34 ± 1.29	0.178 ± 0.150	7.9 ± 3.1	4.80 ± 2.06	0.027 ± 0.025	27% ± 8%
	Nov	8.41 ± 0.14	10 ± 0.2	31.1 ± 0.8	9.79 ± 0.69	0.042 ± 0.049	2.2 ± 1.6	3.13 ± 1.61	0.080 ± 0.577	20% ± 7%
Cage	May	8.10 ± 0.05	16.2 ± 0.6	30.9 ± 0.2	9.26 ± 0.29	0.013 ± 0.005	2.3 ± 1.6	2.54 ± 1.88	0.800 ± 0.996	79% ± 12%
	Aug	7.97 ± 0.06	24.3 ± 0.4	31.0 ± 0.2	8.24 ± 0.72	0.032 ± 0.014	2.5 ± 1.3	1.93 ± 1.10	0.053 ± 0.027	77% ± 16%
	Nov	8.06 ± 0.03	13.0 ± 0.1	31.2 ± 0.0	8.16 ± 0.22	0.014 ± 0.003	3.3 ± 1.4	4.26 ± 1.33	0.042 ± 0.008	100% ± 0%

SAL: salinity; DO: dissolved oxygen; MRP: molybdate-reactive phosphorus; COD: chemical oxygen demand; DIN: dissolved inorganic nitrogen.

**Table 3:** Eigenvalues, variance contribution and accumulated contribution rate of principal components analysis (PCA).

Sampling Type	Principal Component	Eigenvalue	Variance Contribution	Accumulated Contribution Rate
Pond culture	1	2.86	31.79	31.79
	2	1.88	20.88	52.68
	3	1.27	14.14	66.82
Cage culture	1	3.16	35.08	35.08
	2	2.32	25.82	60.90
	3	1.09	12.09	82.99

**Table 4:** Various factors loadings in principal components.

Factor	Pond Aquaculture			Cage Aquaculture			
	PC1	PC2	PC3		PC1	PC2	PC3
**pH**	−0.52	−0.37	−0.33		0.71	0.01	0.39
**SAL**	−0.86	−0.10	−0.09		0.24	0.87	−0.28
T	−0.04	0.78	0.13		−0.76	−0.30	−0.26
**DO**	0.03	0.90	0.16		−0.01	0.32	0.83
**COD**	0.72	0.26	0.27		0.03	0.55	0.36
**Chla**	0.74	−0.22	−0.10		0.80	−0.19	−0.22
**MRP**	0.33	0.52	0.10		−0.57	−0.21	−0.63
**DIN**	−0.29	0.37	−0.76		0.47	−0.74	0.21
**ARGs**	−0.02	0.27	0.90		0.57	0.68	−0.06

**Table 5:** Spearman’s correlation coefficient between the factors and principal components (PCs).

Factor	Pond Aquaculture			Cage Aquaculture		
	PC1	PC2	PC3	PC1	PC2	PC3
pH	−0.56 **	−0.47 *	0.33	0.69 **	0.04	−0.45 *
T	−0.17	0.73 **	0.05	−0.75 **	−0.61 **	0.09
SAL	−0.86 **	−0.15	0.15	0.27	0.85 **	0.25
DO	0.02	0.91 **	0.19	0.02	0.40	0.83 **
MRP	0.27	0.57 **	0.00	−0.49 *	−0.18	0.58 **
COD	0.71 **	0.40 *	−0.32	0.10	0.56 **	−0.40
Chla	0.74 **	−0.22	0.03	0.77 **	0.16	0.17
DIN	−0.26	0.23	0.65 **	0.19	−0.40	−0.59 **
ARGs	0.13	0.38 *	−0.82 **	0.61 **	0.83 **	0.07

Note: * and ** show significant differences (*p* < 0.05 and *p* < 0.01, respectively).
